# Two susceptible *HLA-DRB1* alleles for multiple sclerosis differentially regulate anti-JC virus antibody serostatus along with fingolimod

**DOI:** 10.1186/s12974-020-01865-7

**Published:** 2020-07-09

**Authors:** Mitsuru Watanabe, Yuri Nakamura, Noriko Isobe, Masami Tanaka, Ayako Sakoda, Fumie Hayashi, Yuji Kawano, Ryo Yamasaki, Takuya Matsushita, Jun-ichi Kira

**Affiliations:** 1 Department of Neurology, Neurological Institute, Graduate School of Medical Sciences, Kyushu University, 3-1-1 Maidashi, Higashi-ku, Fukuoka, 812-8582 Japan; 2 Department of Neurology, Brain and Nerve Center, Fukuoka Central Hospital, International University of Health and Welfare, 2-6-11 Yakuin, Chuo-ku, Fukuoka, 810-0022 Japan; 3 School of Pharmacy at Fukuoka, International University of Health and Welfare, 137-1 Enokizu, Okawa, 831-8501 Japan; 4 Department of Neurological Therapeutics, Neurological Institute, Graduate School of Medical Sciences, Kyushu University, 3-1-1 Maidashi, Higashi-ku, Fukuoka, 812-8582 Japan; 5 Kyoto MS Center, Kyoto Min-Iren-Chuo Hospital, 2-1 Uzumasatsuchimoto-cho, Ukyo-ku, Kyoto, 616-8147 Japan; 6 Department of Neurology, Kaikoukai Jyousai Hospital, 1-4 Kitabatake, Nakamura-ku, Nagoya, 453-0815 Japan; 7 Department of Neurology, National Hospital Organization Omuta National Hospital, 1044-1 Oaza, Tachibana, Omuta, 837-0911 Japan; 8 Translational Neuroscience Center, Graduate School of Medicine, and School of Pharmacy at Fukuoka, International University of Health and Welfare, 137-1 Enokizu, Okawa, 831-8501 Japan

**Keywords:** Fingolimod, JC virus, Human leukocyte antigen (HLA), Multiple sclerosis, Progressive multifocal leukoencephalopathy

## Abstract

**Background:**

Progressive multifocal leukoencephalopathy (PML) caused by JC virus (JCV) is a rare but serious complication of some disease-modifying drugs used to treat multiple sclerosis (MS). Japanese MS patients treated with fingolimod were reported to be 10 times more likely to develop PML than equivalent patients in other countries. The strongest susceptibility *human leukocyte antigen* (*HLA*) class II alleles for MS are distinct between races (DRB1*15:01 for Caucasians and DRB1*04:05 and DRB1*15:01 for Japanese); therefore, we investigated whether *HLA* class II alleles modulate anti-JCV antibody serostatus in Japanese MS patients with and without fingolimod.

**Methods:**

We enrolled 128 Japanese patients with MS, in whom 64 (50%) were under fingolimod treatment at sampling, and examined the relationship between *HLA* class II alleles and anti-JCV antibody serostatus. Serum anti-JCV antibody positivity and index were measured using a second-generation two-step assay and *HLA-DRB1* and *-DPB1* alleles were genotyped.

**Results:**

HLA-DRB1*15 carriers had a lower frequency of anti-JCV antibody positivity (57% vs 78%, *p* = 0.015), and lower antibody index (median 0.42 vs 1.97, *p* = 0.037) than non-carriers. Among patients without HLA-DRB1*15, DRB1*04 carriers had a higher seropositivity rate than non-carriers (84% vs 54%, *p* = 0.030), and DPB1*04:02 carriers had a higher anti-JCV antibody index than non-carriers (3.20 vs 1.34, *p* = 0.008) although anti-JCV antibody-positivity rates did not differ. Patients treated with fingolimod had a higher antibody index than other patients (1.46 vs 0.64, *p* = 0.039) and treatment period had a positive correlation with antibody index (*p* = 0.018). Multivariate logistic regression analysis revealed that age was positively associated, and HLA-DRB1*15 was negatively associated with anti-JCV antibody positivity (odds ratio [OR] = 1.06, *p* = 0.006, and OR = 0.37, *p* = 0.028, respectively). Excluding HLA-DRB1*15-carriers, DRB1*04 was an independent risk factor for the presence of anti-JCV antibody (OR = 5.50, *p* = 0.023).

**Conclusions:**

HLA-DRB1*15 is associated with low anti-JCV antibody positive rate and low JCV antibody index, and in the absence of DRB1*15, DRB1*04 carriers are associated with a high antibody positive rate in Japanese, suggesting the effects of two susceptible *HLA-DRB1* alleles on anti-JCV antibody serostatus differ.

## Background

Progressive multifocal leukoencephalopathy (PML) is a rare but frequently fatal demyelinating disease caused by JC virus (JCV) [1–3]. In PML, pathogenic variants of JCV invade the brain either within B cells or as cell-free virus, and infect oligodendrocytes, leading to the apoptosis of infected oligodendrocytes and multifocal demyelination, particularly under conditions of reduced immune surveillance in the central nervous system [1–4]. Some disease-modifying drugs (DMDs) for multiple sclerosis (MS) can cause PML [5]. Following the long-term usage of natalizumab, MS patients with anti-JCV antibody, particularly those with a high antibody index, have a high risk of developing PML [6–8]. Although the incidence rate of fingolimod-associated PML was estimated to be low (0.056–0.069 per 1000 individuals) worldwide, especially in Europe and the USA [5, 9], 4 Japanese patients on fingolimod have developed PML to date, and the incidence rate of fingolimod-associated PML in Japan is estimated to be 10 times higher (0.58–0.65 per 1000) than other countries [10–12]. However, the reason for this remains to be elucidated.

A previous study reported that Scandinavian and German MS patients with anti-JCV antibodies had a significantly lower frequency of the *human leukocyte antigen* (*HLA*)-DRB1*15 haplotype than those without anti-JCV antibodies, suggesting the DRB1*15 haplotype is negatively associated with anti-JCV antibody positivity [13]. A recent Spanish study revealed that older age increased anti-JCV antibody positivity while HLA-DRB1*15:01 carriers had marginally lower anti-JCV antibody positivity rates than DRB1*15:01 non-carriers (*p* = 0.056) [14]. These findings suggest that anti-JCV antibody serostatus are influenced by *HLA* class II alleles.

Although the detailed mechanism of JCV clearance by a host remains to be elucidated, the immune control of JCV mostly depends on cellular immunity [2–4]. Although high titres of anti-JCV antibodies do not prevent the development of PML [4, 15–18], they can be used as a predictive marker for natalizumab-PML in MS patients [6–8]. It is postulated that host CD4^+^ T cells recognizing JCV antigens secrete proinflammatory cytokines that upregulate HLA class I molecules on JCV-infected cells, and that the presentation of viral antigens by HLA class I molecules promotes CD8^+^ cytotoxic T cells to eliminate JCV-infected cells [19]. This CD4^+^ T cell response varies according to *HLA* class II alleles [20], resulting in distinct forms of JCV clearance. Because high levels of anti-JCV antibodies might reflect the high replicative activity of JCV under poor viral clearance whereas negative or low JCV antibody levels reflect strong viral clearance [13, 21–24], differences in CD4^+^ T cell responses by *HLA* class II alleles might be related to anti-JCV antibody serostatus.

The genetic backgrounds of MS differ between Caucasians and Japanese. Although *HLA*-DRB1*15:01 is strongly associated with MS in Europeans and Japanese, DRB1*04:05, which is rare in Caucasians, is also a frequent and strong genetic risk factor in Japanese [25–29]. Therefore, we hypothesized that distinct disease-susceptible *HLA* class II alleles between Caucasian and Japanese patients with MS are associated with differences in JCV clearance and anti-JCV antibody serostatus, which may in part be related to the difference in risk for fingolimod-PML between Caucasian and Japanese patients. Because DNA from Japanese patients with fingolimod-PML was not available for *HLA* genotyping, we studied the relationship between anti-JCV antibody serostatus and *HLA* class II alleles in Japanese MS patients with and without fingolimod to assess whether MS-susceptible *HLA* class II alleles influenced anti-JCV antibody serostatus. The results of the current study suggest why fingolimod-PML risk is higher in Japanese patients compared with Caucasian patients.

## Methods

### Participants

We enrolled 128 Japanese patients with MS in this study. Patients were recruited from the Department of Neurology at Kyushu University Hospital (Fukuoka, Japan), Kyoto Min-Iren-Chuo Hospital (Kyoto, Japan) and Kaikoukai Jyousai Hospital (Nagoya, Japan). A diagnosis of MS was based on the 2010 McDonald criteria [30]. Medical records and laboratory data of patients were retrospectively reviewed. Disease severity was evaluated using Kurtzke’s Expanded Disability Status Scale (EDSS) [31]. Serum samples were collected between 1 July 2014 and 31 December 2018 and stored at − 80 °C at each hospital.

### Standard protocol approval, registration, and patient consent

This study was reviewed and approved by the Ethical Committee of Kyushu University (approved number 575–08). All patients provided written informed consent.

### Measurement of anti-JCV antibody

Serum anti-JCV antibody serostatus and index were determined using a second-generation two-step assay [32] performed at Focus Diagnostics (Cypress, CA, USA).

### *HLA* class II genotyping

Genotyping of the *HLA-DRB1* and *-DPB1* alleles of participants was performed by hybridization between polymerase chain reaction amplification products of the genes and sequence-specific oligonucleotide probes as described previously [33].

### Statistics

Categorical variables were described by counts and percentages, and continuous and ordinal variables by median and interquartile ranges (IQRs) (and range, if necessary). Demographic features were compared between anti-JCV antibody-positive and antibody-negative groups using Fisher’s exact test or the Wilcoxon test. Anti-JCV antibody frequency and index were also compared between two groups using Fisher’s exact test and the Wilcoxon test, respectively. In addition to the comparison of anti-JCV antibody frequency and index between patients with and without each *HLA* allele (shown as four-digits), we also compared them between *HLA* allele groups (shown as two-digits), which reflect HLA serotypes, on the basis of their similar function in each allele group. The associations between anti-JCV antibody index and age, months of treatment with fingolimod, and number of lymphocytes were tested using linear regression models. The linear dose effect of each *HLA* allele on anti-JCV antibody seropositivity was evaluated with the Cochran–Armitage trend test. A multivariable logistic regression model was used to identify independent factors that predicted the positivity of anti-JCV antibody. All analyses were performed using JMP® Pro version 14.1.0 software (SAS Institute, Cary, NC, USA). The significance level was set at *p* <  0.05.

## Results

### Demographics and anti-JCV antibody status of participants

The demographics and anti-JCV antibody status of participants are shown in Table 1. Of 128 patients with MS, 83 (64.8%) had a history of fingolimod usage and 64 (50.0%) were under treatment with fingolimod at sampling. Eighty-six participants (67.2%) were positive for anti-JCV antibody and the median anti-JCV antibody index was 1.19 (IQR = 0.21–3.09).

**Table 1:** Demographic features and anti-JCV antibody status of study participants

	MS patients (*n* = 128)
Sex, female	93 (72.7%)
Age, years	39 [IQR 32–48; range 20–70]
Disease duration, years	10 [IQR 5–16; range 0–43]
EDSS score	2.0 [IQR 1.0–3.5; range 0–8.0]
Lymphocyte counts at sampling, /μL	709 [IQR 447–1539; range 222–3491]
History of fingolimod usage	83 (64.8%)
Use of fingolimod at sampling^a^	64 (50.0%)
Anti-JCV antibody, positive	86 (67.2%)
Anti-JCV antibody index	1.19 [IQR 0.21–3.09; range 0.06–4.04]

Values indicate the median [IQR and range] or count (%)

*EDSS* Expanded Disability Status Scale, *IQR* interquartile range, *JCV* JC virus, *MS* multiple sclerosis

^a^Other disease-modifying drugs were used in 39 patients (30.5%) at sampling: interferon-β-1a in 15 (11.7%), dimethyl fumarate in 13 (10.2%), glatiramer acetate in 4 (3.1%), natalizumab in 3 (2.3%), interferon-β-1b in 2 (1.6%), azathioprine in 1 (0.8%), and methotrexate in 1 (0.8%)

### *HLA* class II alleles in participants

Phenotype frequencies of the *HLA* class II of participants are shown in Additional file 1: Table S1. By four-digit alleles, 51 participants (39.8%) carried HLA-DRB1*04:05 and 42 (32.8%) had DRB1*15:01. By two-digit alleles, 81 (63.3%) had HLA-DRB1*04 and 65 (50.8%) had DRB1*15. The phenotype frequencies were not significantly different between MS patients with and without fingolimod treatment (Additional file 1: Table S1).

### Age is positively associated with anti-JCV antibody positivity and index

**Fig. 1:**
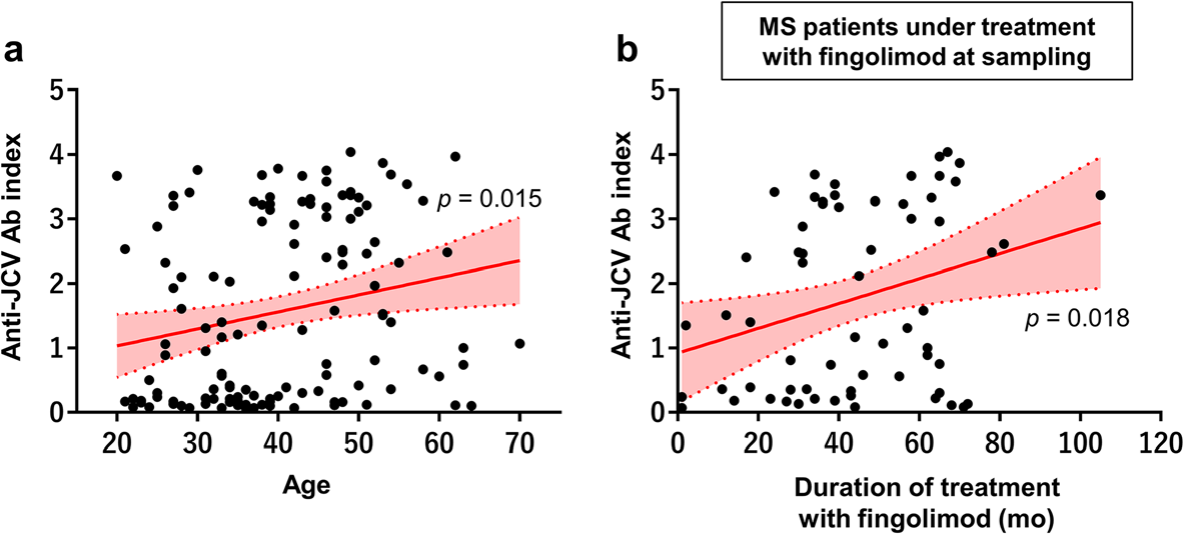
Correlation of anti-JCV antibody index and clinical parameters in patients with MS. **a** Correlation between anti-JCV antibody index and age in all participants (*n* = 128). **b** Correlation between anti-JCV antibody index and duration of treatment with fingolimod in MS patients under fingolimod treatment at sampling (*n* = 64). The lines represent the linear regression of the data. Translucent bands indicate 95% confidence intervals. *p* values were obtained using linear regression analyses. Ab = antibody; JCV = JC virus; mo = months; MS = multiple sclerosis

Next, we assessed the association between anti-JCV antibody serostatus and patients’ demographic and clinical features. Anti-JCV antibody-positive patients were older than antibody-negative patients (*p* = 0.001; Table 2) and age was positively, but weakly, associated with anti-JCV antibody index (*r* = 0.214, *p* = 0.015; Fig. 1a). Although lymphocyte counts were similar between anti-JCV antibody-positive and antibody-negative groups, they tended to have a weak negative association with the anti-JCV antibody index (*r* = − 0.169, *p* = 0.057). Sex, disease duration, and EDSS scores did not differ by anti-JCV antibody serostatus and were not associated with the anti-JCV antibody index.

**Table 2:** Comparison of the demographic features and anti-JCV antibody status between anti-JCV antibody-positive and -negative patients with MS

	Positive (*n* = 86)	Negative (*n* = 42)	*p* value
Sex, female	60 (69.8%)	33 (78.6%)	0.399
Age, years	43.5 [33.0–50.3]	35.0 [27.8–40.3]	0.001
Disease duration, years	11 [5–18]	9 [5–15]	0.455
EDSS score	2.0 [1.0–4.1]	2.0 [1.0–3.5]	0.576
Lymphocyte counts at sampling, /μL	645 [427–1402]	1158 [521–1589]	0.139
Anti-JCV antibody index	2.47 [1.15–3.29]	0.16 [0.11–0.21]	< 0.001

Values indicate the median [IQR] or count (%)

*EDSS* Expanded Disability Status Scale, *IQR* interquartile range, *JCV* JC virus

### Duration of fingolimod treatment is positively associated with anti-JCV antibody index

When comparing anti-JCV antibody serostatus between patients with and without a history of fingolimod treatment (*n* = 83 and 45, respectively), no significant differences were observed for the positivity rate or anti-JCV antibody index. However, patients with fingolimod treatment at sampling (*n* = 64) had a higher antibody index than those without (*n* = 64) (1.46 [0.34–3.26] vs 0.64 [0.16–2.61], *p* = 0.039; Table 3). Duration of treatment with fingolimod was positively associated with the anti-JCV antibody index in patients treated with fingolimod at sampling (*r* = 0.296, *p* = 0.018; Fig. 1b), but not in patients with a history of fingolimod treatment (*r* = 0.204, *p* = 0.066). The positive correlation between duration of treatment with fingolimod and anti-JCV antibody index was still significant in patients under fingolimod treatment at sampling after correcting for age (*p* = 0.022).

**Table 3:** Comparison of the anti-JCV antibody status between MS patients with and without fingolimod treatment

History of fingolimod treatment	Yes (*n* = 83)	No (*n* = 45)	*p* value
Anti-JCV antibody, positive	59 (71.1%)	27 (60.0%)	0.239
Anti-JCV antibody index	1.40 [0.26–3.18]	0.57 [0.16–2.97]	0.129
Fingolimod treatment at sampling	Yes (*n* = 64)	No (*n* = 64)	*p* value
Anti-JCV antibody, positive	47 (73.4%)	39 (60.9%)	0.187
Anti-JCV antibody index	1.46 [0.34–3.26]	0.64 [0.16–2.61]	0.039

Values indicate the median [IQR] or count (%)

*IQR* interquartile range, *JCV* JC virus, *MS* multiple sclerosis

### Relationship between anti-JCV serostatus and *HLA* class II alleles

**Fig. 2:**
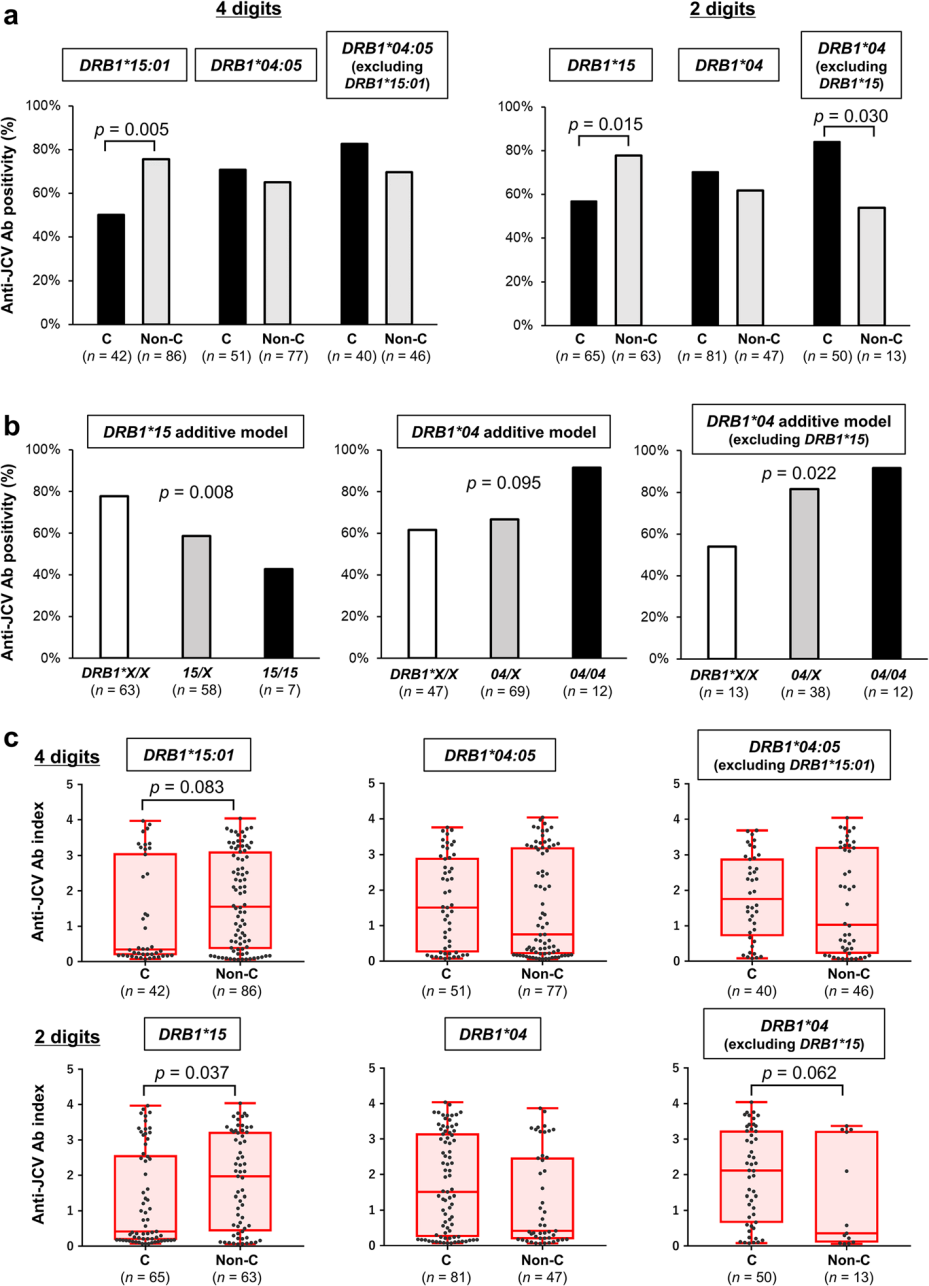
Relationship between anti-JCV antibody serostatus and *HLA* class II alleles in Japanese MS patients. **a** Anti-JCV antibody-positive rates were compared between carriers and non-carriers of each *HLA-DRB1* allele. The *HLA-DRB1* allele was analyzed and is shown as 4 digits in the left panel and as 2 digits in the right panel. *p* values were obtained using Fisher’s exact test. **b** The relationships between anti-JCV antibody seropositivity rates and number of HLA-DRB1*15 or DRB1*04 alleles are shown. X in the figure indicates other alleles specified in each panel (other than DRB1*15 in the left panel and other than DRB1*04 in the middle and right panels). *p* values were obtained using the Cochran–Armitage trend test. **c** Anti-JCV antibody indices were compared between carriers and non-carriers of each *HLA-DRB1* allele. *HLA-DRB1* alleles were analyzed and are shown as 4 digits in the upper panels and as 2 digits in the lower panels. Boxes depict the median and interquartile ranges, and upper/lower whiskers extend from the hinge toward the largest/smallest values. *p* values were obtained using the Wilcoxon test. Ab = antibody; C = carriers; *HLA* = *human leukocyte antigen*; JCV = JC virus; MS = multiple sclerosis; non-C = non-carriers

When we analyzed *HLA* class II by four-digit alleles, HLA-DRB1*15:01 carriers (*n* = 42) had a lower anti-JCV antibody-positive rate than DRB1*15:01 non-carriers (50% vs 76%, *p* = 0.005; Fig. 2a). However, the positive rate of anti-JCV antibody was not different between HLA-DRB1*04:05 carriers (*n* = 51) and non-carriers (71% vs 65%, *p* = 0.567). When analyzing *HLA* class II by two-digit alleles, HLA-DRB1*15 carriers (*n* = 65) had a lower positivity rate of anti-JCV antibody compared with DRB1*15 non-carriers (57% vs 78%, *p* = 0.015; Fig. 2a). Again, anti-JCV antibody positive rates were similar between HLA-DRB1*04 carriers (*n* = 81) and non-carriers (70% vs 62%, *p* = 0.335). However, when we excluded HLA-DRB1*15 carriers because DRB1*15 had a very strong effect on anti-JCV antibody status, DRB1*04 carriers (*n* = 50) had a higher anti-JCV antibody positive rate compared with DRB1*04 non-carriers (*n* = 13) (84% vs 54%, *p* = 0.030; Fig. 2a). Anti-JCV antibody positivity rates were estimated to decrease per HLA-DRB1*15 allele (*p* = 0.008) and to increase per DRB1*04 allele when excluding DRB1*15 (*p* = 0.022), which suggests a gene-dosage effect (Fig. 2b). In addition, in the fingolimod-treated group, HLA-DRB1*15:01 carriers (*n* = 22) had a significantly lower positivity rate of anti-JCV antibody compared with DRB1*15:01 non-carriers (50% vs 86%, *p* = 0.006; Additional file 2: Figure S1a). Analyzing *HLA* class II by two-digits, HLA-DRB1*15 carriers (*n* = 32) tended to have a lower positivity rate of anti-JCV antibody compared with DRB1*15 non-carriers (63% vs 84%, *p* = 0.088). When HLA-DRB1*15 carriers were excluded, DRB1*04 carriers (*n* = 24) tended to have a higher anti-JCV antibody positive rate compared with DRB1*04 non-carriers (*n* = 8) (92% vs 63%, *p* = 0.085; Additional file 2: Figure S1a). Such associations between *HLA* alleles and anti-JCV antibody positivity rates were not seen in MS patients without fingolimod treatment (Additional file 2: Figure S1b).

The anti-JCV antibody index tended to be lower in HLA-DRB1*15:01 carriers compared with DRB1*15:01 non-carriers (median 0.35 vs 1.56, *p* = 0.083) and was lower in DRB1*15 carriers than in non-carriers (0.42 vs 1.97, *p* = 0.037, Fig. 2c). Moreover, in HLA-DRB1*15 non-carriers, DRB1*04 carriers tended to have a higher anti-JCV index compared with DRB1*04 non-carriers (2.12 vs 0.36, *p* = 0.062, Fig. 2c).

In addition to HLA-DRB1*04:05 and DRB1*15:01, DRB1*04:10 carriers (*n* = 8) had a higher anti-JCV antibody index compared with non-carriers (3.29 vs 1.03, *p* = 0.021). Of HLA-DRB1*15:01 non-carriers, DPB1*04:02 carriers (*n* = 13) had a higher anti-JCV antibody index compared with non-carriers (*n* = 64) (3.20 vs 1.34, *p* = 0.008). However, anti-JCV antibody-positive rates did not differ between these allele carriers and non-carriers. No other *HLA* class II alleles were associated with anti-JCV antibody serostatus.

### Age and HLA-DRB1*15 are independently associated with anti-JCV antibody serostatus by multivariate analyses

To identify independent factors that predict anti-JCV antibody seropositivity, we performed multivariate logistic regression analysis. It showed that age was positively, and HLA-DRB1*15 was negatively, associated with anti-JCV antibody seropositivity (odds ratio [OR] = 1.06, *p* = 0.006 and OR = 0.37, *p* = 0.028, respectively; Table 4). In HLA-DRB1*15 non-carriers (*n* = 63), DRB1*04 was an independent risk factor for possessing anti-JCV antibodies by multivariate logistic regression analysis (OR = 5.50, *p* = 0.023; Table 5). In both analyses, fingolimod treatment itself was not an independent factor for anti-JCV antibody seropositivity. Moreover, we conducted another multivariate analysis in patients under fingolimod treatment (*n* = 64). Again, age and HLA-DRB1*15 were independent risk factors for anti-JCV antibody seropositivity (OR = 1.42, *p* = 0.002, and OR = 0.02, *p* = 0.022, respectively; Additional file 1: Table S2); however, treatment duration or lymphocyte count were not independent risk factors (*p* = 0.880 and 0.094, respectively; Additional file 1: Table S2).

**Table 4:** Multivariate logistic regression analysis of factors contributing to anti-JCV antibody positivity in patients with MS

All participants (*n* = 128)	OR (95% CI)	*p* value
Age	1.06 (1.02–1.10)	0.006
HLA-DRB1*15	0.37 (0.16–0.90)	0.028
Sex (male)	1.59 (0.63–4.00)	0.321
Fingolimod usage at sampling	1.76 (0.52–5.89)	0.361
Lymphocyte count (× 1000/μL)	1.14 (0.47–2.79)	0.771
HLA-DRB1*04	0.92 (0.39–2.19)	0.850

*CI* confidence interval, *HLA human leukocyte antigen*, *JCV* JC virus, *MS* multiple sclerosis, *OR* odds ratio

**Table 5:** Multivariate logistic regression analysis of factors contributing to anti-JCV antibody positivity in patients with MS excluding carriers of HLA-DRB1*15

Patients without HLA-DRB1*15 (*n* = 63)	OR (95% CI)	*p* value
HLA-DRB1*04	5.50 (1.26–23.94)	0.023
Age	1.04 (0.98–1.11)	0.149
Fingolimod usage at sampling	2.40 (0.33–17.32)	0.384
Sex (male)	1.78 (0.38–8.46)	0.467
Lymphocyte count (× 1000/μL)	0.85 (0.20–3.66)	0.832

*CI* confidence interval, *HLA human leukocyte antigen*, *JCV* JC virus, *MS* multiple sclerosis, *OR* odds ratio

## Discussion

The current study of Japanese patients with MS disclosed that HLA-DRB1*15 was associated with low anti-JCV antibody positivity and low antibody index, and that in the absence of DRB1*15, DRB1*04 carriers were associated with high antibody seropositivity. Moreover, HLA-DRB1*15 allele and DRB1*04 allele without DRB1*15 demonstrated a gene-dosage effect on JCV antibody positivity. Additionally, ageing was positively associated with JCV antibody positivity and index while long-term fingolimod usage increased the JCV antibody index even after adjusting for age.

In addition to the strong negative association of HLA-DRB1*15 with JCV antibody positivity and index, we clearly showed the HLA-DRB1*15 allele had negative gene-dose effects and the DRB1*04 allele had positive gene-dose effects on anti-JCV antibody positivity. These findings indicate the influence of these gene alleles on anti-JCV antibody serostatus. This study is the first to report a lower JCV antibody index in HLA-DRB1*15-positive MS patients compared with DRB1*15-negative patients, which suggests that anti-JCV antibody production is decreased in DRB1*15 carriers. The higher frequency of JCV antibody positivity in HLA-DRB1*04 carriers was observed only after excluding DRB1*15 carriers, which suggests the dominance of HLA-DRB1*15 over DRB1*04 for anti-JCV antibody serostatus.

The findings of this study suggest that the immune control of JCV might be partly related to the strong negative association of HLA-DRB1*15 with JCV antibody positivity and index. However, the detailed mechanism of JCV elimination by the host remains to be elucidated. Although JCV-specific antibody titres are elevated in PML patients [15–17, 19], intrathecal or serum anti-JCV antibodies do not prevent the development of PML. Most individuals who persistently shed JCV in their urine are seropositive [6], some seropositive individuals are viremic [17], and some individuals with PML have high copy numbers of viral DNA despite very high concentrations of anti-JCV antibodies in their cerebrospinal fluid (CSF) [17, 19]. Furthermore, a high JCV antibody index (> 0.9) was strongly associated with PML in natalizumab-treated MS patients [6–8]. These observations collectively indicate that high levels of anti-JCV antibodies are not related to JCV clearance, but rather reflect the high amount of JCV and predict the risk of developing PML. Therefore, immune control of JCV is mainly dependent on cell-mediated immunity [2–4]. It was hypothesized that upon recognition of the viral capsid protein, VP-1, CD4^+^ T cells produce interferon-γ, which upregulates HLA class I molecules on JCV-infected cells. Presentation of the viral large T cell antigen by HLA class I molecules activates CD8^+^ T cells, which remove JCV-infected cells via granzyme B and other cytotoxic molecules [19]. The findings that PML tends to develop in idiopathic CD4^+^ T lymphocytopenia [34], that HIV-seropositive patients with PML lack CD4^+^ T cell responses to JCV [35], and PML patients with low numbers of CSF CD4^+^ T cells remain persistently JCV-positive in the CSF [36] collectively suggest the crucial role of CD4^+^ T cells as well as CD8^+^ cytotoxic T cells in the elimination of JCV and prevention of PML. Indeed, increased numbers of JCV-specific CD4^+^ T cells were found in the peripheral blood of PML survivors [15, 35]. This CD4^+^ T cell response varies according to HLA class II molecules that present the viral antigens [20]. Therefore, differences in CD4^+^ T cell responses related to distinct *HLA* class II alleles are thought to influence the efficacy of JCV clearance [13, 20].

The negative association between HLA-DRB1*15 and anti-JCV antibodies suggests HLA-DRB1*15 carriers present JCV antigens to CD4^+^ T cells to activate CD8^+^ cytotoxic T cell responses that eliminate JCV. Eradication of JCV may result in negative or low anti-JCV antibody serostatus in HLA-DRB1*15 carriers. By contrast, HLA-DRB1*04 carriers were reported to have very low T cell responses to JCV compared with HLA-DRB1*15 carriers [20]. Therefore, reduced T cell responses against JCV might be related to inefficient viral antigen presentation by HLA-DRB1*04 molecules that results in poor viral elimination and enhanced viral replication, which stimulates B cells to produce high levels of anti-JCV antibodies [13, 20, 22, 24]. The low JCV antibody levels in HLA-DRB1*15 carriers and high JCV antibody levels in HLA-DRB1*04 carriers excluding HLA-DRB1*15 carriers found in this study might be explained by this theory.

Although HLA-DRB1*15 is a common susceptibility gene for MS in Caucasians and Japanese, the phenotype frequency of DRB1*15:01 ranges between 45% and 60% in European MS patients (10–30% in the general population) but is only approximately 30% in Japanese patients (16–17% in the general population) [26, 27, 29, 37–39]. Conversely, the phenotype frequency of HLA-DRB1*04:05 was reported to be 0.7% in MS patients in Italy [37], and 0–5.3% in the European general population [40], but up to 25% in the Japanese general population and around 45% in Japanese MS patients [27, 29]. Anti-JCV antibody seropositivity rates in MS are higher in Japanese (approximately 70% in our and previous studies) than in those of European descent (50%–60% in Europe and the USA) [41–43]. This difference might be partly explained by the difference in proportions of JCV-resistant DRB1*15:01 and JCV-susceptible DRB1*04:05 alleles between these two races. Thus, the high frequency of fingolimod-PML in Japanese may in part be related to these common susceptibility *HLA* genes. This possibility should be verified in future large-scale studies.

In fingolimod users, ageing is a risk and DRB1*15:01 is a strong resistance factor for anti-JCV antibody positivity. Ageing is a risk for anti-JCV antibody positivity in Europeans and Japanese [41–44]. The increase of JCV antibody index levels with the long-term usage of fingolimod, which was significant even after correcting for age, is consistent with a previous study in Japanese [42]. Fingolimod acts as a functional antagonist of the sphingosine 1-phosphate receptor, which results in the inhibition of central memory T cell egress from lymph nodes [45]. This effect is thought to be its beneficial mode of action in MS [45]. Although the precise mechanism of fingolimod-PML is still poorly defined, T cell surveillance in the central nervous system may be dampened upon fingolimod usage. The finding that lower lymphocyte counts tended to be associated with a higher anti-JCV antibody index in our MS patients may support this mechanism. It was established that a high JCV antibody index (> 0.9) was a risk for developing PML in natalizumab-treated MS patients [8]. Although it remains to be established whether a high JCV antibody index is also a risk factor for fingolimod-PML, our study results suggest that MS patients with these risk factors, namely, aged, non-DRB1*15:01 carriers with anti-JCV antibody, should be frequently monitored for JCV antibody index and PML lesions by MRI upon long-term usage of fingolimod. Additionally, DMDs that lack PML risk, such as first line injectables, may be recommended for DRB1*04 carriers with MS because DRB1*04:05-positive MS patients, especially Japanese patients, have a relatively mild disease course [27, 29].

There were several limitations in our study. First, a high anti-JCV antibody index is not a proven risk factor for developing PML associated with fingolimod treatment. Thus, although our study showed the differential effects of two *HLA* class II susceptible alleles for MS related to anti-JCV antibody serostatus, it does not necessarily mean that these alleles are susceptibility or resistance genes for PML development itself in MS patients treated with fingolimod. Because only four patients with fingolimod-PML have been reported in Japan, none of whom were at our institute [11, 12], we could not determine the *HLA* alleles in fingolimod-PML patients. Second, we did not examine JCV DNA in blood, primarily because its presence in blood is not a reliable predictor of PML. JCV DNA is often not detected in the blood of PML patients whereas it is occasionally detected in the blood of HIV-positive patients without PML [46]. Furthermore, natalizumab-treated patients with detectable JCV DNA in the blood (< 1%) did not develop PML with continued natalizumab treatment, and JCV DNA was not detectable in blood collected prior to PML development in natalizumab-treated PML patients [47]. Therefore, investigating the association between anti-JCV antibody serostatus and *HLA* alleles might help us to understand the immune responses against JCV and resultant risk of PML in MS patients. Third, we did not examine T cell response to JCV peptides. Because stock sera are much easier to collect than live T cells, we investigated the correlation between anti-JCV antibodies and *HLA* alleles. Because our data suggest that differences in the immunogenetic background among races are at least partly related to anti-JCV antibody serostatus, T cell responses to JCV peptides should be examined as the next step to elucidate differences in the defense mechanism against JCV by *HLA* alleles in MS patients. Fourth, we showed data for *HLA* as four- and two-digits in this study. This was based on the hypothesis that all alleles in each allele group have a similar function. However, some alleles in the same allele group can present different antigen peptides and will therefore have different T cell activation capabilities [20]. Future large-scale studies are needed to identify whether the effects on anti-JCV serostatus are similar among four-digit *HLA* alleles belonging to identical two-digit allele groups. Fifth, we did not genotype other *HLA* alleles such as *HLA-DQB1*. Because previous studies showed that the HLA-DQB1*06:03 haplotype was also positively associated with JCV-serostatus [13] and that phenylalanine at position 9 of *HLA-DQB1* was associated with MS susceptibility in Japanese [48], susceptible *HLA* alleles other than *HLA-DRB1* and *-DPB1* may also be related to the immune response against JCV in patients with MS. Therefore, future studies should assess the association between other *HLA* alleles and JCV serostatus. Finally, our study was a cross-sectional study enrolling a relatively small number of MS patients because of the rarity of MS in Japanese. The low median EDSS scores in this study cohort might reflect the milder disability of Japanese patients with MS compared with European patients with a similar disease duration and age [49, 50]. Therefore, we think that the relatively low EDSS scores in our patients are not necessarily related to selection bias. However, our findings should be regarded as preliminary and confirmed by large-scale longitudinal studies.

## Conclusions

HLA-DRB1*15:01 and DRB1*15 alleles are associated with low JCV antibody positive rate and index, whereas DRB1*04 is associated with high JCV antibody positive rate in Japanese MS patients, excluding DRB1*15 carriers. Therefore, the two MS-susceptible *HLA-DRB1* alleles exert distinct effects on anti-JCV antibody serostatus in Japanese patients with MS.

## Supplementary information

**Additional file 1: Table S1.** Phenotype frequencies of *HLA* class II alleles in study participants. **Table S2.** Multivariate logistic regression analysis of factors contributing to anti-JCV antibody positivity in patients with MS under fingolimod treatment.

**Additional file 2: Figure S1.** Relationship between anti-JCV antibody serostatus and *HLA* class II alleles in Japanese MS patients with and without fingolimod. (a, b) Anti-JCV antibody-positive rates were compared between carriers and non-carriers of each *HLA-DRB1* allele in MS patients with (a) and without (b) fingolimod treatment. The *HLA-DRB1* allele was analyzed and is shown as 4 digits in the left panel and as 2 digits in the right panel. *p* values were obtained using Fisher’s exact test. Ab = antibody; C = carriers; *HLA* = *human leukocyte antigen*; JCV = JC virus; MS = multiple sclerosis; Non-C = non-carriers.

## Data Availability

The datasets generated and/or analyzed during the present study will be available from the corresponding author based on the guidelines of the Ethics Committee of Kyushu University upon reasonable request from any qualified investigator.

## Abbreviations

CSF: Cerebrospinal fluid
DMD: Disease-modifying drug
EDSS: Expanded Disability Status Scale
HLA: Human leukocyte antigen
IQR: Interquartile range
JCV: JC virus
MS: Multiple sclerosis
OR: Odds ratio
PML: Progressive multifocal leukoencephalopathy
