# Supplementary material for: Two susceptible HLA-DRB1 alleles for multiple sclerosis differentially regulate anti-JC virus antibody serostatus along with fingolimod
Source: J Neuroinflammation. 2020 Jul 9;17:206. doi: 10.1186/s12974-020-01865-7 (PMC7350631; doi:10.1186/s12974-020-01865-7)
Supplement: Supplementary file 1 — Additional file 1: Table S1. Phenotype frequencies of HLA class II alleles in study participants. Table S2. Multivariate logistic regression analysis of factors contributing to anti-JCV antibody positivity in patients with MS under fingolimod treatment. [file 12974_2020_1865_MOESM1_ESM.pdf]

Table S1 Phenotype frequencies of *HLA* class II alleles in MS patients with and without fingolimod

DRB1	MS patients with fingolimod (*n* = 64)	MS patients without fingolimod (*n* = 64)	*DPB1*	MS patients with fingolimod (*n* = 59)[a]	MS patients without fingolimod (*n* = 56)[a]
01:01	2 (3.1)	3 (4.7)	*01:01*	1 (1.7)	0 (0)
04:01	0 (0)	1 (1.6)	*02:01*	17 (28.8)	12 (21.4)
04:03	7 (10.9)	6 (9.4)	*02:02*	3 (5.1)	5 (8.9)
04:04	1 (1.6)	0 (0)	*03:01*	7 (11.9)	10 (17.9)
04:05	31 (48.4)	20 (31.3)	*04:01*	4 (6.8)	3 (5.4)
04:06	7 (10.9)	8 (12.5)	*04:02*	10 (16.9)	9 (16.1)
04:10	2 (3.1)	6 (9.4)	*05:01*	43 (72.9)	33 (58.9)
07:01	1 (1.6)	0 (0)	*06:01*	1 (1.7)	0 (0)
08:02	6 (9.4)	3 (4.7)	*09:01*	11 (18.6)	14 (25.0)
08:03	8 (12.5)	5 (7.8)	*13:01*	2 (3.4)	1 (1.8)
09:01	7 (10.9)	16 (25.0)	*14:01*	2 (3.4)	3 (5.4)
11:01	0 (0)	2 (3.1)	*17:01*	1 (1.7)	0 (0)
12:01	1 (1.6)	5 (7.8)	*19:01*	0 (0)	1 (1.8)
12:02	1 (1.6)	2 (3.1)	*25:01*	0 (0)	2 (3.6)
13:01	0 (0)	2 (3.1)	*36:01*	0 (0)	1 (1.8)
13:02	3 (4.7)	1 (1.6)	*41:01*	1 (1.7)	0 (0)
14:03	5 (7.8)	0 (0)			
14:05	3 (4.7)	1 (1.6)			
14:06	2 (3.1)	2 (3.1)			
14:54	1 (1.6)	3 (4.7)			
15:01	22 (34.4)	20 (31.3)			
15:02	12 (18.8)	16 (25.0)			
16:02	2 (3.1)	2 (3.1)			

Values indicate count (%).

[a]No data were available for the *HLA-DPB1* allele in 5 patients with fingolimod and 8 patients without fingolimod.

The frequency of each allele was not significantly different between patients with and without fingolimod treatment.

HLA = human leukocyte antigen; MS = multiple sclerosis.

Table S2 Multivariate logistic regression analysis of factors contributing to anti-JCV antibody positivity in patients with MS under fingolimod treatment

**Patients under fingolimod treatment (*n* = 64)**	**OR (95% CI)**	***p* value**
Age	1.42 (1.14–1.77)	0.002
*HLA-DRB1*15*	0.02 (0.00–0.57)	0.022
Lymphocyte count (×1,000/μL)	171.0 (0.42–69,526.1)	0.094
*HLA-DRB1*04*	3.59 (0.39–32.99)	0.258
Sex (male)	0.55 (0.06–5.46)	0.609
Duration of treatment with fingolimod (y)	1.04 (0.59–1.84)	0.880

CI = confidence interval; *HLA = human leukocyte antigen*; JCV = JC virus; MS = multiple sclerosis; OR = odds ratio; y = years.
