# Supplementary material for: Two susceptible HLA-DRB1 alleles for multiple sclerosis differentially regulate anti-JC virus antibody serostatus along with fingolimod
Source: J Neuroinflammation. 2020 Jul 9;17:206. doi: 10.1186/s12974-020-01865-7 (PMC7350631; doi:10.1186/s12974-020-01865-7)
Supplement: Supplementary file 2 — Additional file 2: Figure S1. Relationship between anti-JCV antibody serostatus and HLA class II alleles in Japanese MS patients with and without fingolimod. (a, b) Anti-JCV antibody-positive rates were compared between carriers and non-carriers of each HLA-DRB1 allele in MS patients with (a) and without (b) fingolimod treatment. The HLA-DRB1 allele was analyzed and is shown as 4 digits in the left panel and as 2 digits in the right panel. p values were obtained using Fisher’s exact test. Ab = antibody; C = carriers; HLA = human leukocyte antigen; JCV = JC virus; MS = multiple sclerosis; Non-C = non-carriers. [file 12974_2020_1865_MOESM2_ESM.pdf]

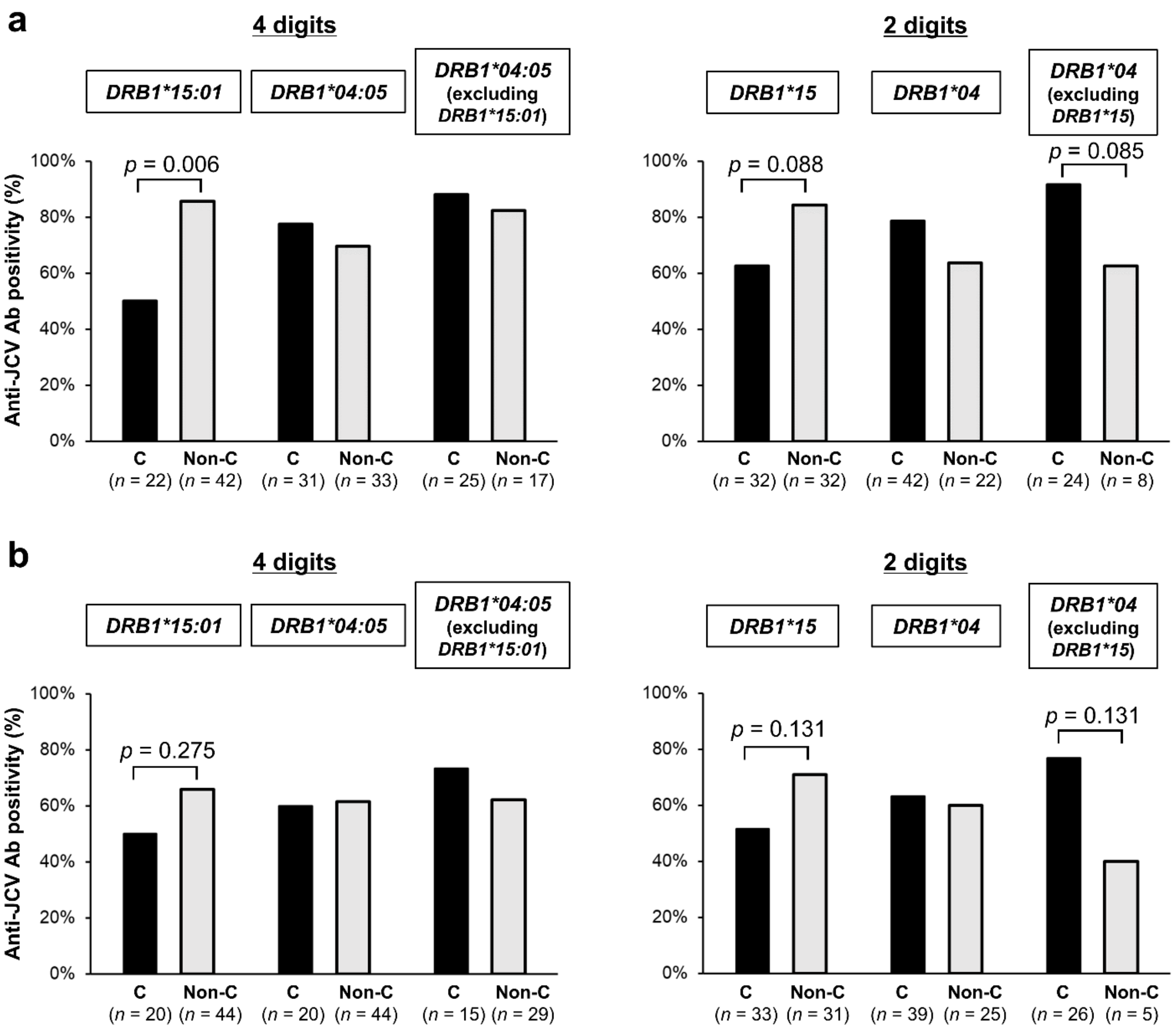

Fig. S1 Relationship between anti-JCV antibody serostatus and *HLA* class II alleles in Japanese MS patients with and without fingolimod.

(**a**, **b**) Anti-JCV antibody-positive rates were compared between carriers and non-carriers of each *HLA-DRB1* allele in MS patients with (**a**) and without (**b**) fingolimod treatment. The *HLA-DRB1* allele was analysed and is shown as 4 digits in the left panel and as 2 digits in the right panel. *p* values were obtained using Fisher's exact test.

Ab = antibody; C = carriers; *HLA* = *human leukocyte antigen*; JCV = JC virus; MS = multiple sclerosis; Non-C = non-carriers.
